# Association of the *hOGG1* Ser326Cys polymorphism with gynecologic cancer susceptibility: a meta-analysis

**DOI:** 10.1042/BSR20203245

**Published:** 2020-11-26

**Authors:** Yongzhong Shi, Wei Xu, Xia Zhang

**Affiliations:** 1 Institute of Translational Medicine, Hunan Provincial People’s Hospital, the First Affiliated Hospital of Hunan Normal University, Changsha, Hunan, China; 2 Hunan Maternal and Child Health Hospital, Changsha, Hunan, China

**Keywords:** gynecologic cancer, hOGG1, meta-analysis, Ser326Cys, susceptibility

## Abstract

The association between the *hOGG1* Ser326Cys polymorphism and gynecologic cancer susceptibility is inconclusive. We performed a comprehensive meta-analysis to precisely estimate of the impact of the *hOGG1* Ser326Cys polymorphism on gynecologic cancer susceptibility. Electronic databases including PubMed, Embase, WanFang, and the China National Knowledge Infrastructure were searched for relevant studies. Odds ratios (ORs) with corresponding 95% confidence intervals (CIs) were determined to assess the strength of the association. Fourteen studies with 2712 cases and 3638 controls were included in the final meta-analysis. The pooled analysis yielded a significant association between the *hOGG1* Ser326Cys polymorphism and overall gynecologic cancer susceptibility (dominant model: OR = 1.16, 95% CI = 1.03–1.30, *P*=0.017). A significantly higher gynecologic cancer risk was found for the European population (homozygous model: OR = 2.17, 95% CI = 1.80–2.61, *P*<0.001; recessive model: OR = 2.11, 95% CI = 1.41–3.17, *P*<0.001; dominant model: OR = 1.29, 95% CI = 1.12–1.48, *P*<0.001; and allele model: OR = 1.40, 95% CI = 1.13–1.74, *P*=0.002), but not in the Asian population. The stratified analysis by cancer type revealed endometrial cancer was significantly associated with the *hOGG1* Ser326Cys polymorphism (dominant model: OR = 1.29, 95% CI = 1.09–1.54, *P*=0.003; and allele model: OR = 1.28, 95% CI = 1.02–1.60, *P*=0.031). In conclusion, the *hOGG1* Ser326Cys polymorphism was associated with higher overall gynecologic cancer susceptibility, especially for endometrial cancer in the European population.

## Introduction

Gynecologic cancer, including cervical, ovarian, and endometrial cancer, is the major cause of cancer-related deaths in women worldwide. Among them, cervical cancer is the fourth leading cause of deaths with an estimated 311,000 deaths in 2018 worldwide, followed by 185,000 deaths from ovarian cancer, and over 89,000 deaths from endometrial cancer [1,2]. Consequentially, gynecological cancer has become a serious threat to the health and lives of women around the world. Thus, it is necessary to identify the exact molecular mechanisms underlying gynecologic carcinogenesis.

DNA damage can initiate the genetic instability that drives cancer development [3,4]. DNA damage can be induced by various mechanisms such as by-products of endogenous normal metabolism or exposure to environmental mutagens [5]. Thus, it is very important to repair this damage to maintain genetic stability against cancer-causing agents. Several DNA repair pathways exist and perform different roles in repairing different types of DNA damage [6,7]. Of these, the base excision repair (BER) pathway plays a key role in handling small base lesions in DNA resulting from oxidation and alkylation damage by specific DNA glycosylase [8]. As a major product of oxidative DNA damage, 8-oxoguanine can be excised from DNA by the human 8-oxoguanine DNA glycosylase1 (hOGG1) enzyme, which is a vital member of the BER pathway [9].

The *hOGG1* gene is located on chromosome 3p26.2 and encodes a glycosylase that catalyzes the excision of 8-oxoguanine adducts from damaged DNA [10]. In *hOGG1* knockout mice, 8-oxoguanine was found to accumulate in the genomic DNA and carcinoma developed spontaneously [11]. Furthermore, genetic polymorphisms in the *hOGG1* gene have been confirmed to exert different activities in the repair of 8-oxoguanine in the complementation assay of defective *Escherichia coli* variant, carrying, for example, the *hOGG1* Ser326Cys (rs1052133C>G) polymorphism [12]. To date, many studies have investigated the association between the *hOGG1* Ser326Cys polymorphism and gynecologic cancer risk. However, there are still many inconsistencies. An increased risk was found by some studies [13–17], but was not confirmed by others [18–26]. Thus, a meta-analysis is a good approach to investigate the association between the *hOGG1* Ser326Cys polymorphism and the susceptibility to gynecologic cancer. To date, only one meta-analysis evaluated the association with overall gynecologic cancer using subgroup analysis and included only five studies published before September 2014 [27]. Nonetheless, there has been no meta-analysis that has specifically focused on gynecologic cancer types, such as cervical, ovarian, and endometrial cancer. In addition, several additional studies on gynecologic cancer have been published since 2014 [14,15,18]. Thus, the aim of the present study was to perform an updated meta-analysis investigating the association between the *hOGG1* Ser326Cys polymorphism and susceptibility to gynecologic cancer.

## Materials and methods

### Literature search

Relevant studies were retrieved from electronic databases including PubMed, Embase, WanFang, and the China National Knowledge Infrastructure using the following search terms: ‘hOGG1 or OGG1’, ‘polymorphism or variant or variation’, and ‘cancer or tumor or carcinoma’. We also evaluated previously published meta-analyses and review articles for other relevant studies. The language was limited to English and Chinese. All the relevant articles were searched before August 1, 2020.

### Inclusion and exclusion criteria

The inclusion criteria were set as follows: (1) case–control study design evaluating the association between the *hOGG1* Ser326Cys polymorphism and susceptibility to gynecologic cancer and (2) available information about genotype and allele frequency for genetic model analysis. The exclusion criteria were as follows: (1) no case–control studies; (2) reviews, case reports, meta-analyses, and letters and (3) studies with insufficient data. In addition, studies with genotype frequencies in the control groups deviating from Hardy–Weinberg equilibrium (HWE) were also included in our meta-analysis if further evidence showed that other polymorphisms did not deviate from HWE.

### Data extraction and quality assessment

According to the inclusion and exclusion criteria listed above, two authors independently extracted information from all eligible publications and any disagreements were resolved through discussions with other authors. The following information was collected from each included study: first author’s surname, year of publication, country, ethnicity, cancer type, source of control, genotyping method, and genotype frequency. Different ethnicities were categorized as either European or Asian population and different sources of control were defined as hospital-based (HB) and population-based (PB). The quality of each study was evaluated using quality assessment criteria as outlined in previous studies [28]. The quality score ranged from 0 to 15, with scores 0 to 9 or 10 to 15 considered low or high quality, respectively.

### Trial sequential analysis (TSA)

Trial Sequential Analysis Viewer (TSA) (version 0.9.5.10, Copenhagen Trial Unit, Centre for Clinical Intervention Research, Copenhagen, Denmark) was used to perform the analysis using the data retrieved from the studies. The parameters were set as followed: 5% type I error, 20% relative risk reduction, and 20% type II error (a statistical test power of 80%). If the cumulative *Z*-curve crossed the TSA monitoring boundary or exceeded the required information size, firm evidence had been reached. Otherwise, more studies were needed [29].

### Statistical analysis

The Chi-square test was applied to assess the HWE in the control groups. The strength of the association between the *hOGG1* Ser326Cys polymorphism and the susceptibility to gynecologic cancer was evaluated by calculating the odds ratios (ORs) with their corresponding 95% confidence intervals (CIs). The pooled ORs were obtained for the homozygote model (Cys/Cys vs. Ser/Ser), heterozygote model (Ser/Cys vs. Ser/Ser), dominant model (Ser/Cys + Cys/Cys vs. Ser/Ser), recessive model (Cys/Cys vs. Ser/Cys + Ser/Ser), and the allele model (Cys vs. Ser). Stratified analyses were also carried out for ethnicity, cancer type, source of control, and genotyping method. The Chi-square-based *Q* test was used to assess the degree of heterogeneity among studies, and a *P*-value < 0.10 was regarded as significant. If no heterogeneity existed with *P* > 0.10, the fixed-effect model was used [30]. Otherwise, the random-effect model was applied [31]. Sensitivity analysis was performed by sequentially removing a single study each time from the analysis to test the stability of the results. The funnel plot and Egger’s liner regression test were adopted to detect the potential publication bias. Moreover, we performed a false-positive report probability (FPRP) analysis to evaluate all the significant findings, and set 0.2 as the FPRP threshold [32,33]. Only the results with FPRP values less than 0.2 were considered significant findings. Expression quantitative trait loci (eQTL) analysis in the GTEx portal (https://www.gtexportal.org/home/) was used to evaluate the correlation between the *hOGG1* Ser326Cys polymorphism and levels of mRNA expression of hOGG1. All statistical tests were performed using STATA version 11.0 (STATA Corporation, College Station, TX, U.S.A.).

## Results

### Study characteristics

A total of 498 relevant articles were retrieved from the electronic database search. Overall, 480 publications were excluded after title and abstract screening. Of the remaining 18 publications, one study was excluded because of insufficient genotype information for data analysis [34]; one study was excluded because it did not involve the *hOGG1* Ser326Cys polymorphism [35], and two studies were excluded because the genotype frequencies in the control groups were not consistent with HWE and also other evidence showed that other polymorphisms did not satisfied HWE [13,36]. Overall, 14 studies with 2712 cases and 3638 controls were included in the final meta-analysis [14–26,37]. The study characteristics are shown in Table 1. Data from four publications were found to deviate from HWE [14,17,19,25], we decided to include these based on further evidence that other polymorphisms satisfied HWE. Among the 14 studies, there were 7 endometrial cancer studies, 4 ovarian cancer studies, and 3 cervical cancer studies. Nine studies involved the European population and 5 studies involved the Asian population. Eight studies were PB and six were HB designs, respectively. The genotyping method adopted by most studies was polymerase chain reaction-restriction fragment length polymorphism (PCR-RFLP). Furthermore, 6 studies were considered as high quality and 8 were considered as low quality.

**Table 1:** Characteristics of studies included in this meta-analysis

Surname	Year	Country	Ethnicity	Cancer type	SC	Method	Cases				Controls				MAF	HWE	Score
							Total	CC	CG	GG	Total	CC	CG	GG			
Smolarz	2018	Poland	European	Endometrial	HB	PCR-RFLP	610	160	160	290	610	196	230	184	0.49	<0.001	11
Hosono	2013	Japan	Asian	Endometrial	PB	RT-PCR	91	30	40	21	261	77	112	72	0.49	0.022	10
Sobczuk	2012	Poland	European	Endometrial	PB	PCR-RFLP	94	64	23	7	114	83	28	3	0.15	0.731	8
Cincin	2012	Turkey	European	Endometrial	HB	PCR-RFLP	104	57	45	2	158	111	41	6	0.17	0.375	8
Romanowicz- Makowska	2011	Poland	European	Endometrial	HB	PCR-RFLP	150	94	46	10	150	105	39	6	0.17	0.335	7
Krupa	2011	Poland	European	Endometrial	PB	PCR-RFLP	30	23	6	1	30	22	7	1	0.15	0.462	8
Attar	2010	Turkey	European	Endometrial	PB	PCR-RFLP	52	35	15	2	101	70	27	4	0.17	0.501	8
Verma ^#^	2019	India	Asian	Ovarian	PB	PCR-RFLP	130	/	/	/	150	/	/	/	/	/	9
Michalska	2015	Poland	European	Ovarian	HB	PCR-RFLP	720	160	160	400	720	196	340	184	0.49	0.138	10
Chen	2011	China	Asian	Ovarian	PB	PCR-RFLP	420	81	176	163	840	144	446	250	0.56	0.022	12
Arcand	2005	Canada	European	Ovarian	HB	SSCP	91	57	24	10	57	30	22	5	0.28	0.739	10
Xiong	2010	China	Asian	Cervical	PB	PCR-RFLP	86	17	45	24	102	24	45	33	0.54	0.263	9
Farkasova	2008	Slovakia	European	Cervical	PB	PCR-RFLP	18	10	7	1	25	10	15	0	0.50	0.032	8
Niwa	2005	Japan	Asian	Cervical	HB	PCR-RFLP	116	37	60	19	320	94	146	80	0.48	0.125	10

Abbreviations: HB, hospital-based; HWE, Hardy–Weinberg equilibrium; MAF, minor allele frequency; PB, population-based; PCR-RFLP, polymerase chain reaction-restriction fragment length polymorphism; RT-PCR, real-time PCR; SC, source of control; SSCP, single-strand conformation polymorphism.

# This study would be included to calculate the association under allele model.

### Meta-analysis results

**Figure 1:**
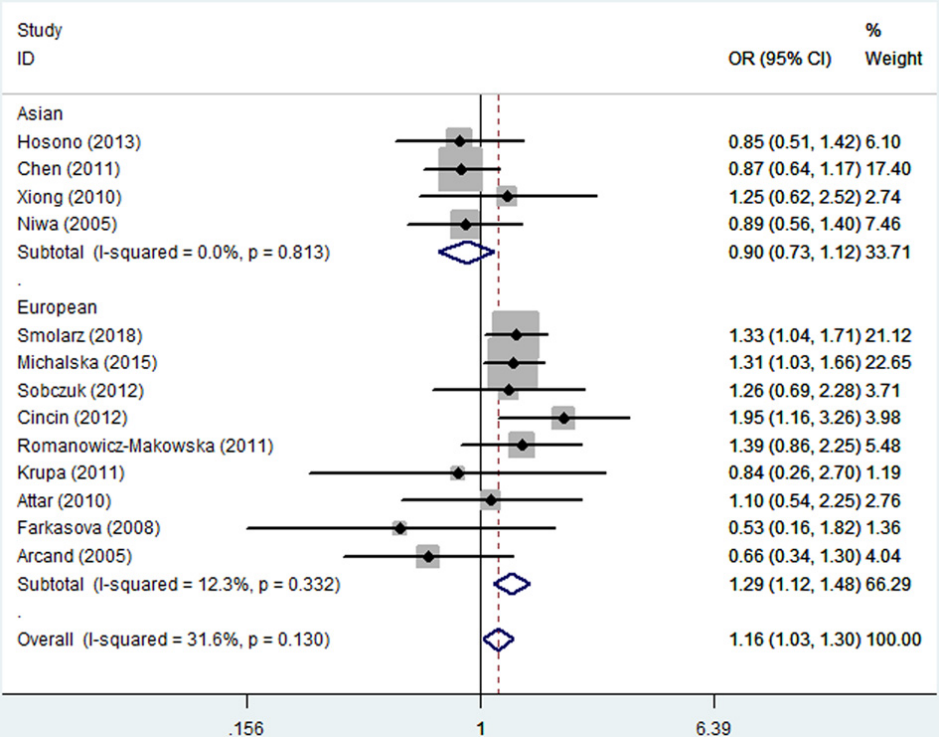
Forest plot of the association between the *hOGG1* Ser326Cys polymorphism and gynecologic cancer susceptibility in the stratified analysis by ethnicity under the dominant model

**Figure 2:**
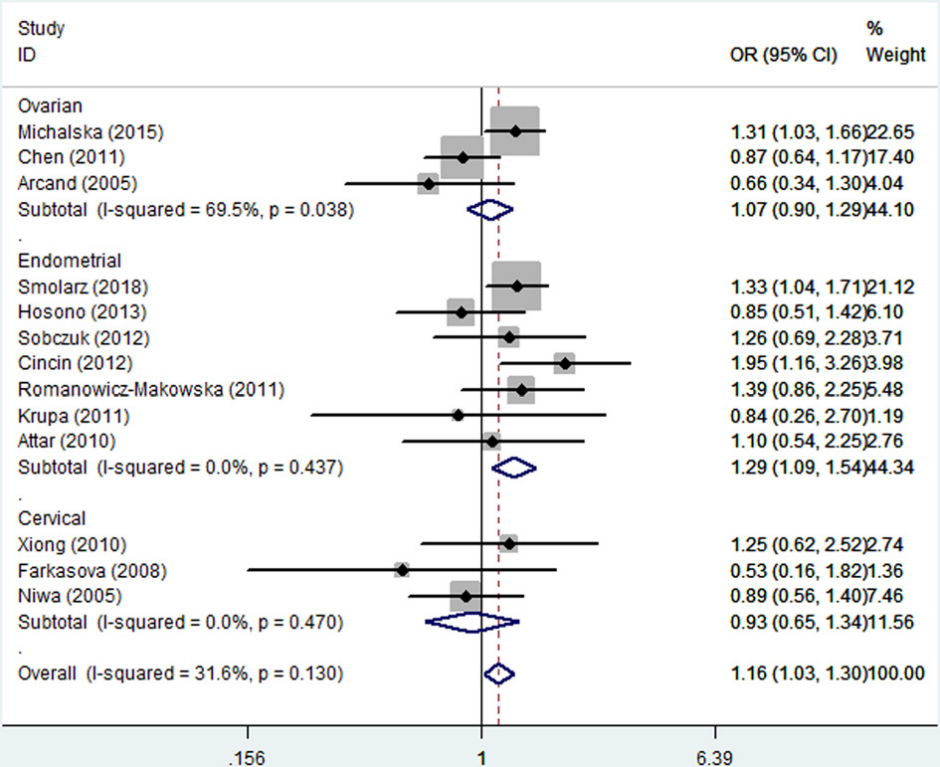
Forest plot of the association between the *hOGG1* Ser326Cys polymorphism and gynecologic cancer susceptibility in the stratified analysis by cancer type under the dominant model

As listed in Table 2, the pooled analysis yielded a significant association between the *hOGG1* Ser326Cys polymorphism and increased susceptibility to gynecologic cancer (dominant model: OR = 1.16, 95% CI = 1.03–1.30). In the stratified analysis by ethnicity, a significantly increased gynecologic cancer risk was found in the European population (homozygous model: OR = 2.17, 95% CI = 1.80–2.61; recessive model: OR = 2.11, 95% CI = 1.41–3.17; dominant model: OR = 1.29, 95% CI = 1.12–1.48; and allele model: OR = 1.40, 95% CI = 1.13–1.74, Figure 1), but not in the Asian population. In the stratified analysis by cancer type, a statistically significant association was identified among cases of ovarian cancer (heterozygous model: OR = 0.62, 95% CI = 0.51–0.76) and endometrial cancer (dominant model: OR = 1.29, 95% CI = 1.09–1.54; and the allele model: OR = 1.28, 95% CI = 1.02–1.60, Figure 2). The stratified analysis by the source of the control and genotyping method also revealed a significant association was found among these subgroups (Table 2).

**Table 2:** Meta-analysis of the association between *hOGG1* Ser326Cys polymorphism and gynecologic cancer susceptibility

Various	*N*	Homozygous		Heterozygous		Recessive		Dominant		Allele	
		OR (95% CI)	*P*^het^	OR (95% CI)	*P*^het^	OR (95% CI)	*P*^het^	OR (95% CI)	*P*^het^	OR (95% CI)	*P*^het^
**Total**	14	1.31 (0.93–1.86)	<0.001	0.93 (0.67–1.29)	0.004	1.36 (0.91–2.04)	<0.001	1.16 (1.03–1.30)	0.130	1.12 (0.90–1.39)	<0.001
**Ethnicity**											
**Asian**	5	0.92 (0.67–1.26)	0.272	0.89 (0.67–1.17)	0.274	0.90 (0.55–1.48)	0.004	0.90 (0.73–1.12)	0.813	0.89 (0.71–1.12)	0.027
**European**	9	2.17 (1.80–2.61)	0.447	0.93 (0.67–1.29)	0.002	2.11 (1.41–3.17)	0.078	1.29 (1.12–1.48)	0.332	1.40 (1.13–1.74)	0.004
**Cancer type**											
**Ovarian**	4	1.60 (0.80–3.21)	0.001	0.62 (0.51–0.76)	0.651	2.06 (0.97–4.37)	<0.001	1.07 (0.90–1.29)	0.038	1.09 (0.66–1.79)	<0.001
**Endometrial**	7	1.40 (0.89–2.21)	0.147	1.13 (0.86–1.48)	0.123	1.36 (0.80–2.31)	0.034	1.29 (1.09–1.54)	0.437	1.28 (1.02–1.60)	0.048
**Cervical**	3	0.76 (0.46–1.24)	0.427	1.04 (0.68–1.59)	0.331	0.70 (0.46–1.05)	0.406	0.93 (0.65–1.34)	0.470	0.86 (0.68–1.08)	0.702
**Source of control**											
**PB**	8	1.10 (0.84–1.44)	0.69	0.85 (0.68–1.06)	0.538	1.17 (0.82–1.68)	0.197	0.94 (0.76–1.16)	0.810	0.97 (0.80–1.17)	0.144
**HB**	6	1.46 (0.90–2.37)	0.001	0.96 (0.66–1.40)	<0.001	1.50 (0.83–2.71)	<0.001	1.27 (1.10–1.47)	0.112	1.33 (0.98–1.80)	<0.001
**Genotyping method**											
**PCR-RFLP**	12	1.44 (0.99–2.07)	0.001	0.97 (0.75–1.25)	0.002	1.47 (0.95–2.28)	<0.001	1.20 (1.06–1.36)	0.212	1.18 (0.94–1.49)	<0.001

Abbreviations: HB, hospital-based; PB, population-based; PCR-RFLP, polymerase chain reaction-restriction fragment length polymorphism.

### Heterogeneity and sensitivity analyses

**Figure 3:**
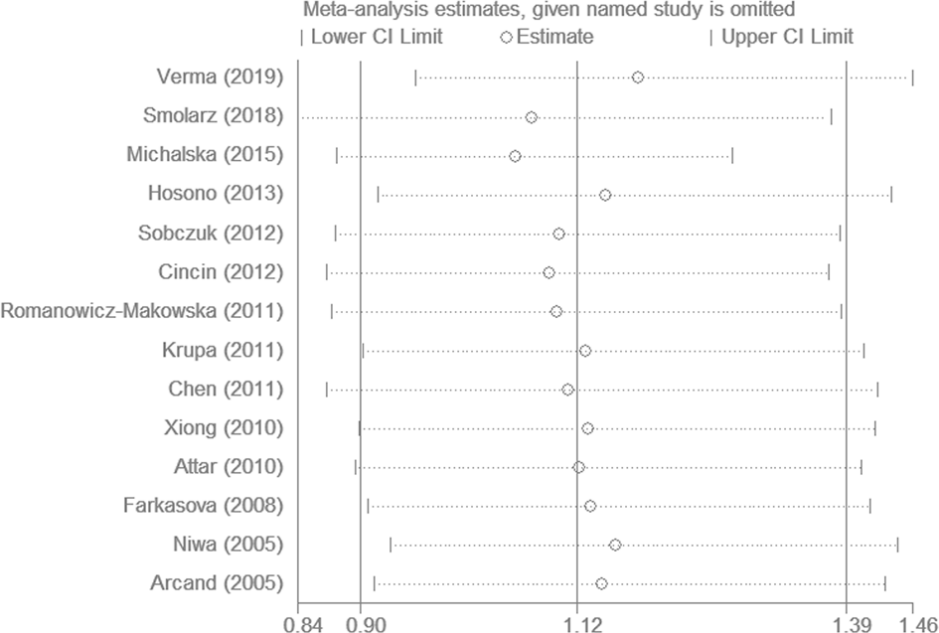
Influence analysis of the summary odds ratio coefficients under the allele model

Substantial heterogeneities were observed among all studies for the association between the *hOGG1* Ser326Cys polymorphism and gynecologic cancer susceptibility (homozygous model: *P*<0.001; heterozygous model: *P*=0.004; recessive model: *P*<0.001, and the allele model: *P*<0.001, Figure 3), except for the dominant model (*P*=0.130). Thus, there was no significant change in the ORs and 95% CIs after recalculations by sequentially removing one single study, suggesting the stability of our results.

### Publication bias

**Figure 4:**
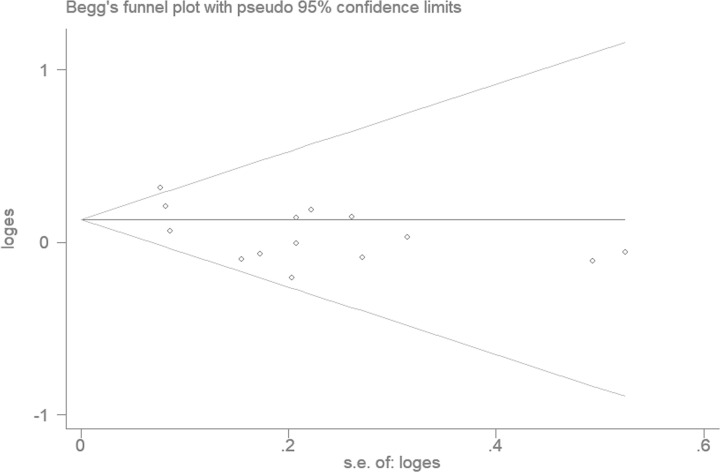
Funnel plot analysis to detect publication bias under the allele model

The funnel plot and Egger’s liner regression test were used to detect a potential publication bias. As shown in Figure 4, the results indicated that there was no evidence of publication bias for any of the models (homozygous model: *P*=0.182, heterozygous model: *P*=0.228, recessive model: *P*=0.149, and dominant model: *P*=0.252), except for the allele model (*P*=0.035).

### Trial sequential analysis results

**Figure 5:**
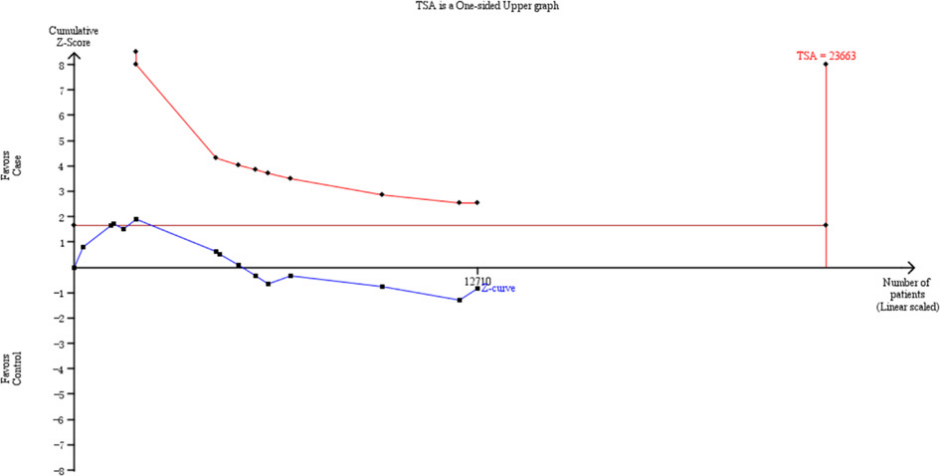
Trial sequential analysis of the association between the *hOGG1* Ser326Cys polymorphism and gynecologic cancer susceptibility under the allele model

As shown in Figure 5, the cumulative *Z*-curve for the *hOGG1* Ser326Cys polymorphism was evaluated using TSA analysis, and showed the pooled studies failed to reach the required information size, which suggested that the cumulative boundary was not achieved and further studies with larger sample sizes would be required to verify these associations.

### False-positive report probability results

We selected 0.2 as the FPRP threshold. As listed in Table 3, at the prior probability of 0.1, all the significant findings remained noteworthy, except for the results regarding endometrial cancer under the allele model.

**Table 3:** False-positive report probability values for associations between *hOGG1* Ser326Cys polymorphism and gynecologic cancer susceptibility

Variables	OR (95% CI)	*P* value	Statistical power	Prior probability				
				0.25	0.1	0.01	0.001	0.0001
**Homozygous**								
**European**	2.17 (1.80–2.61)	<0.001	0.564	0.000	0.000	0.000	0.000	0.000
**Recessive**								
**European**	2.11 (1.41–3.17)	<0.001	0.580	0.002	0.005	0.052	0.358	0.848
**Dominant**	1.16 (1.03–1.30)	0.017	0.720	0.043	0.118	0.595	0.937	0.993
**European**	1.29 (1.12–1.48)	<0.001	0.544	0.002	0.005	0.049	0.340	0.838
**Endometrial**	1.29 (1.09–1.54)	0.003	0.534	0.026	0.075	0.473	0.901	0.989
**HB**	1.27 (1.10–1.47)	0.001	0.623	0.007	0.019	0.178	0.685	0.956
**PCR-RFLP**	1.20 (1.06–1.36)	0.004	0.500	0.025	0.072	0.460	0.896	0.989
**Allele**								
**European**	1.40 (1.13–1.74)	0.002	0.500	0.014	0.042	0.324	0.829	0.980
**Endometrial**	1.28 (1.02–1.60)	0.031	0.554	0.140	0.329	0.843	0.982	0.998

Abbreviations: HB, hospital-based; PCR-RFLP, polymerase chain reaction-restriction fragment length polymorphism.

### Effects of the Ser326Cys polymorphism on the expression of hOGG1

**Figure 6:**
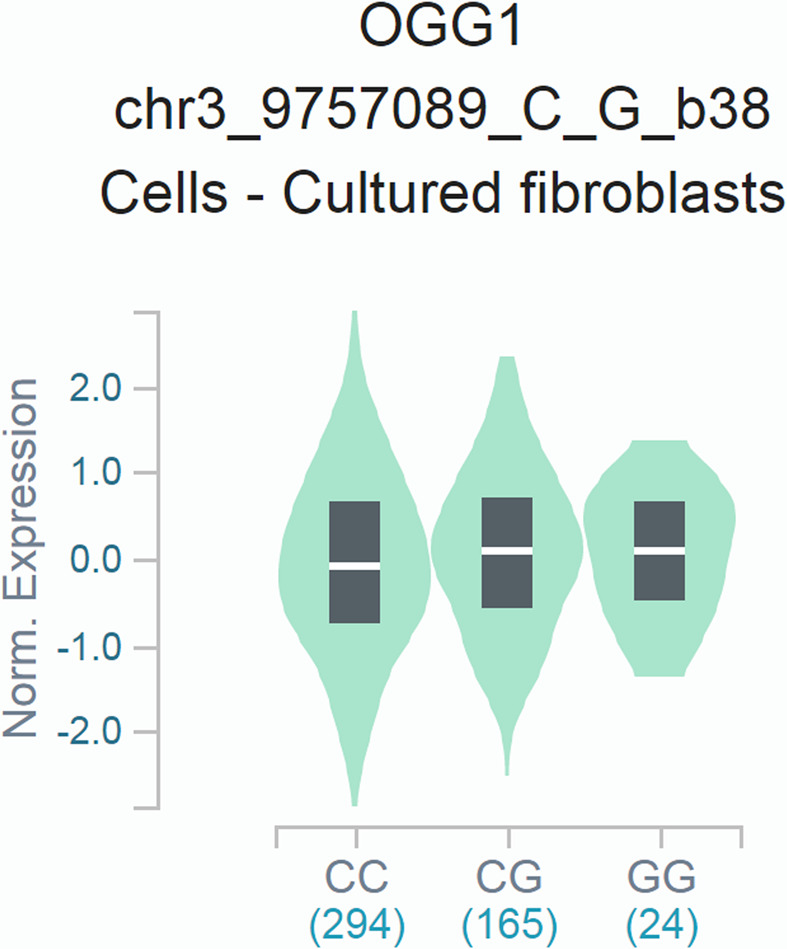
Functional relevance of the *hOGG1* Ser326Cys polymorphism on hOGG1 mRNA expression extracted from the GTEx Database The *hOGG1* 326Cys allele was significantly associated with higher expression of hOGG1 in the fibroblast cell cultures (*P*=2.2 × 10^−5^).

We further assessed the impact of the *hOGG1* Ser326Cys polymorphism on the mRNA expression of hOGG1 using the GTEx web tool. The 326Cys allele was significantly associated with higher expression of hOGG1 in the cultured fibroblasts cells (Figure 6).

## Discussion

Gynecological carcinogenesis is still poorly understood. It is a complex event influenced by environmental and genetic factors, as well as by gene–environment interactions. In recent years, genetic factors are increasingly believed to be important contributors to carcinogenesis, and, the imbalance between DNA damage and repair [38,39]. Over 100 proteins are involved in the DNA repair system, and of these, hOGG1 is a key protein involved in excising and removing 8-oxoguanine adducts from damaged DNA [40,41]. Low hOGG1 protein activity was found to associate with a higher risk of various of cancers [42]. Furthermore, the *hOGG1* Ser326Cys polymorphism, the most studied *hOGG1* polymorphism, was found to influence protein activity, and thus, contribute to carcinogenesis [12]. Currently, many studies have investigated the association between the *hOGG1* Ser326Cys polymorphism and gynecologic cancer susceptibility. However, the results have been conflicting and inconclusive.

To the best of our knowledge, this is the first meta-analysis focusing on the association between the *hOGG1* Ser326Cys polymorphism and gynecologic cancer susceptibility. Overall, the current meta-analysis included 14 studies with 2712 cases of gynecologic cancer and 3638 controls and observed a significant association between the *hOGG1* Ser326Cys polymorphism and overall gynecologic cancer susceptibility. Furthermore, stratified analysis by ethnicity indicated that *hOGG1* Ser326Cys polymorphism was associated with increased gynecologic cancer risk in the European population, but not in the Asian population. Moreover, in the stratified analysis by cancer type, a statistically significant association was identified among ovarian cancer and endometrial cancer, but not with cervical cancer.

Two previous meta-analyses investigated the association between the *hOGG1* Ser326Cys polymorphism and gynecologic cancer susceptibility [27,43]. Both studies investigated the association between overall cancer risk and then performed a stratified analysis for gynecologic cancer. The first study, published in 2015, included only five gynecologic cancer studies and found the *hOGG1* Ser326Cys polymorphism was associated with overall gynecologic cancer susceptibility in a homozygous model [27]. As for our results, we updated the data and obtained a precise estimation of the lack of association of gynecologic cancer risk after including nine additional studies in the homozygous model, but obtained a stronger association only in the dominant model. In the previous meta-analysis, three of the five studies involved endometrial cancer, and one each concerned cervical and ovarian cancer. In our meta-analysis, 7 of the 14 included studies investigated endometrial cancer, 3 studies involved cervical cancer, and 4 ovarian cancer. Additional studies are necessary to verify these results. The second meta-analysis, published in 2016, included only five endometrial cancer studies and observed no association between the *hOGG1* Ser326Cys polymorphism and endometrial cancer [43]. In comparison, our meta-analysis included two additional studies and revealed that the *hOGG1* Ser326Cys polymorphism was associated with an increased risk of endometrial cancer. As expected, there have been a relatively small number of studies that have focused on this specific association. However, we collected all the available studies and provided a comprehensive evaluation of their relationship. Further, we applied TSA to verify the above results, which suggested that additional studies with larger sample sizes would be needed to confirm these associations.

The stratified analysis by cancer type indicated that the *hOGG1* Ser326Cys polymorphism was statistically associated with the susceptibility of ovarian cancer and endometrial cancer, but not of cervical cancer. Furthermore, we conducted a statistical analysis stratifying these three types of gynecologic cancer, as shown in Table 2. Moreover, a statistically significant association with the polymorphism was found for ovarian cancer both in the Asian population (heterozygous model: OR = 0.70, 95% CI = 0.51–0.97) and the European population (heterozygous model: OR = 0.58, 95% CI = 0.44–0.75), and for endometrial cancer in the European population (homozygous model: OR = 1.86, 95% CI = 1.44–2.40; recessive model: OR = 2.01, 95% CI = 1.61–2.50; dominant model: OR = 1.36, 95% CI = 1.14–1.64, allele model: OR = 1.51, 95% CI = 1.33–1.73). However, no association existed for cervical cancer or endometrial cancer in the Asian population. These differences suggested the possible involvement of ethnic differences and tumor tissue specificity, as well as different environment factors [44].

Several limitations of this meta-analysis should be addressed. First, lack of original data limited any additional estimation of gene–gene and gene–environment interactions. Second, the number of cases and controls enrolled in the current meta-analysis was relatively small. Third, notable heterogeneities were observed for several models, thus a random-effect model was applied.

In conclusion, our study revealed that the *hOGG1* Ser326Cys polymorphism was associated with an increased risk of overall gynecologic cancer susceptibility, especially for endometrial cancer in the European population. More eligible case–control studies are necessary to further confirm our findings.

## Data Availability

The data are available from the corresponding author upon reasonable request.

## Abbreviations

BER: base excision repair
FPRP: false-positive report probability
HB: hospital-based
hOGG1: human 8-oxoguanine DNA glycosylase 1
HWE: Hardy–Weinberg equilibrium
MAF: minor allele frequency
PB: population-based
PCR-RFLP: polymerase chain reaction-restriction fragment length polymorphism
RT-PCR: real-time PCR
SC: source of control
SSCP: single-strand conformation polymorphism
TSA: trial sequential analysis
